# Variable-Selection Emerges on Top in Empirical Comparison of Whole-Genome Complex-Trait Prediction Methods

**DOI:** 10.1371/journal.pone.0138903

**Published:** 2015-10-06

**Authors:** David C. Haws, Irina Rish, Simon Teyssedre, Dan He, Aurelie C. Lozano, Prabhanjan Kambadur, Zivan Karaman, Laxmi Parida

**Affiliations:** 1 Computational Biology Center, IBM T. J. Watson Research, Yorktown Heights, NY 10598, United States of America; 2 Business Analytics and Mathematical Sciences Department, IBM T. J. Watson Research, Yorktown Heights, NY 10598, United States of America; 3 Limagrain Europe, Centre de Recherche de Chappes, CS 3911, Route d’Ennezat, Chappes 63720, France; China Agricultural Univeristy, CHINA

## Abstract

Accurate prediction of complex traits based on whole-genome data is a computational problem of paramount importance, particularly to plant and animal breeders. However, the number of genetic markers is typically orders of magnitude larger than the number of samples (*p* >> *n*), amongst other challenges. We assessed the effectiveness of a diverse set of state-of-the-art methods on publicly accessible real data. The most surprising finding was that approaches with feature selection performed better than others on average, in contrast to the expectation in the community that variable selection is mostly ineffective, i.e. that it does not improve accuracy of prediction, in spite of *p* >> *n*. We observed superior performance despite a somewhat simplistic approach to variable selection, possibly suggesting an inherent robustness. This bodes well in general since the variable selection methods usually improve interpretability without loss of prediction power. Apart from identifying a set of benchmark data sets (including one simulated data), we also discuss the performance analysis for each data set in terms of the input characteristics.

## Introduction

Genomic selection (GS) can be viewed as a form of marker-assisted selection (MAS), where a statistical model is trained on available genetic and phenotypic data; a genomic estimated breeding value (GEBV) is then estimated for current or future individuals based only on their genetic data and the trained model. The GEBV can then be used to select favorable individuals. In quantitative genetics, EBV’s were first estimated using a linear regression model, called the *infinitesimal model*, dating far back to [1]. The covariance between individuals in the population were given by a kinship matrix traditionally estimated by pedigree information. In a seminal paper, Meuwissen *et al* [2] showed through simulations that GEBV’s could be accurately estimated from a *marker effects* model using genome wide dense marker data, marking the transition of quantitative genetics to genome-wide regression. The relationship between additive genetic variance of the classical quantitative genetics models and variance of marker effects in regression models is very well expounded by [3]. In the marker effects model the phenotype is modeled by a linear model where each marker has an additive contribution. Around the same time, the infinitesimal model was adapted such that marker data was used to compute the covariance between individuals, and would later be called the *Genomic Best Linear Unbiased Prediction* (GBLUP). In fact, it was later shown by multiple authors that under reasonable assumptions the GBLUP model and marker effects model (specifically rrBLUP) are identical [3–6]. Further studies have confirmed that GS using marker effects is an accurate method for selection, and can often outperform pedigree-based selection even for traits with low heritability [7].

Many parametric as well as non-parametric methods have been proposed to tackle GS using markers, e.g. see [8] for the importance of GS and an overview of some of the most well-known methods. Moreover, the underlying problem that is at the heart of GS—prediction when the number of samples is drastically smaller than the number of variables, i.e., “small *n*, large *p* problem”—is certainly not unfamiliar to the statistics and machine learning communities. Hence, most GS methods build models where either the effect of each marker is forced to be very small or only few markers are allowed to have any effect, or some combination of both limitations is used. For background on GS see [9, 10], and for general background on statistical genetics see [11, 12].

One obvious goal of GS is the practical application to plant and animal breeding programs where in most cases it is more cost effective to genotype new material and estimate GEBV’s as opposed to a lengthy breeding program to evaluate actual phenotypes. Many empirical studies [13, 14] help highlight the myriad of issues that must be tackled in GS, such as high versus low heritability [15], infinitesimal versus marker effects model on mice [16], what is the reference population (i.e. on what data is the statistical model trained) [17, 18], how to handle multiple populations [19], the difference in generations between the reference population and the evaluation population [5], and *linkage disequilibrium* (LD) between markers and QTL, to name but a few. See the aptly titled work of [20] for a guide of many GS methods and on their applications for MAS. Further, [21] shows the usefulness of GS in simulated maize data. However, one must be aware that certain GS methods may work well in some instances while performing poorly in other. As such, an important determination of the performance of GS is the nature of the phenotype being modeled. Complex traits—traits affected by a large number of genes—are often the most important to researchers and breeders. On the spectrum of fewer to many genes affecting the trait, oligogenic traits are on the former end while complex traits (polygenic) are defined as those on the latter end. A few likely examples of complex traits include crop yield, drought resistance, meat quality, disease resistance, and mass. Indeed, there is a general consensus that most traits are influenced by very large number of markers each with small-effect and additionally the prediction of complex traits requires concurrent consideration of large number of markers [8, 22–24]. Additionally, it has been noted in literature that only some but not all findings from simulations are confirmed by real data [8]. Data simulations suffer from the inherent difficulty of having to assume some genetic architecture as well as patterns of inheritance (such as, additive patterns) that could unduly bias the prediction models.

The goal of this analysis was first, to identify a diverse set of publicly available plant and animal real data sets that can serve as a benchmark for the evaluation of such prediction algorithms. Secondly, assess the effectiveness of a diverse set of (state-of-the-art) methods, including parametric and non-parametric models. In particular, we also included variable selection methods in an attempt to assuage the implications of *p* >> *n*.

We identified four publicly available data sets: Rice, Pig, Maize, and QTLMAS 2010 data. The data sets contain both real plant and animal data as well as simulated data (for completeness), with a range of population structure. The Rice dataset [25] contains 31,633 Single-Nucleotide Polymorphism (SNP) variants from 413 accessions of O. sativa, taken from 82 countries and containing 34 phenotypes. From these we selected *pericarp color* and *protein content* as there were indications each was oligogenic and polygenic respectively [25]. We evaluated the original phenotypes as well as phenotypes corrected for population structure. The Pig data [26] contains 52,842 SNPs on 3,534 animals and two of the five available traits were selected. The QTLMAS data, taken from the QTLMAS 2010 Workshop [27], consists of 2,326 sequenced individuals over five generations (F0–F4) with 20 founders, five male and 15 females, with 10,031 biallelic SNPs. Two phenotype traits were available, the first a quantitative trait and the second a binary trait. Lastly, the Maize data [28], used for the European CornFed program, consists of two maize diversity panels with 261 *flint* and *dent* lines, and 29,094 and 30,027 SNPs respectively. The three traits recorded were male flowering time (Tass_GDD6), plant dry matter yield (DM_Yield), and dry matter content (DMC). See Methods section for more details on the data sets.

The marker effects GS methods were chosen to reflect traditional as well as recent promising methods, some taken directly from the machine learning community. The methods applied were Bayes A, Bayes B, Bayes C*π*, rrBLUP, Epistasis-rrBLUP, Elastic Net, Bayesian LASSO, FOBA, ISIS, SVR, mRMR, and PCA FOBA and are very briefly described here. See Methods section for more details on each method. Ridge Regression Best Linear Unbiased Predictor (rrBLUP), Bayes A, Bayes B, and Bayes C*π* are all marker effects models [2], i.e. the phenotype is modeled by a linear model, where each marker has an additive contribution. The rrBLUP method performs a fit similar to linear regression but also estimates the marker effects (random variables) such that the squared-normal (${\left\|\text{β}\right\|}_{2}^{2}$) of the marker effects is minimal, i.e. the marker effects can all be non-zero but small overall. It is widely considered one of the best GS methods yielding good results with low computational requirements. The Bayes A/B/C*π* methods are Bayesian approaches to the marker effects model which allow for each marker’s effect to have its own variance (Bayes A/B), and/or only some markers having non-zero effect (Bayes B/C*π*). Elastic Net [29] again is based on the marker effects model, this time fitting the model and estimating marker effects such that their squared-normal and sum of absolute values(*l*_1_-norm) are minimal ($\lambda {\left\|\text{β}\right\|}_{2}^{2}+(1-\lambda )\left(\sum |\beta_{i}|\right)$), with a *λ* parameter to emphasize one norm over the other. Both LASSO and Ridge Regression are special cases of Elastic Net. Bayesian LASSO [30, 31], another marker effects model, is a Bayesian approach to a LASSO solution: estimating a linear fit while minimizing the *l*_1_-norm ((∑∣*β*_*i*_∣)). The Forward-Backward (FoBa) heuristic [32] uses the marker effects model and greedily picks markers (forward step) to include in the model which minimize the squared error, then trains a model on all features and greedily removes one feature with smallest increase of squared error (backwards step). FoBa iterates between the forward and backwards steps towards a solution. Support Vector Regression (SVR) [33–37] attempts to model the genotype/phenotype relationship by finding a hyperplane (high-dimensional generalization of a 3d-plane) where all the data points (marker data) lay on or at least as close as possible to the hyperplane. The real trick being the data are first mapped to a different high-dimensional space using a kernel, hence the four kernels studied here: linear, polynomial, radial, and sigmoid. Epistasis-rrBLUP is a method developed by the authors which uses the rrBLUP method but in addition to the marker effects we also include all pairwise multiplicative marker interactions, hence the term “epistasis”. With such a large increase in the number of variables, special computational approaches based on high-performance computing were developed to obtain a solution.

Yet another consensus in the community is echoed as “overall, it seems that with long spans of LD and relatively sparse platforms (e.g., 50,000 SNPs) variable selection may not be needed” [8]. Therefore, we also choose three feature selection methods to test the above claim. In this context, feature selection is the preliminary process of selecting a subset of markers on which to train a GS model. ISIS is an Iterative form of Sure Independence Screening (ISIS) [38], where in basic SIS a subsets of markers are selected according to their correlation to the phenotype and a model fit is performed (in our case rrBLUP). In ISIS, the basic SIS step is repeated, where in subsequent steps the residuals of the rrBLUP are treated as the response variable, and features are again selected. In Principal Component Analysis FOBA (PCA FOBA), PCA was used transductively to select a subset of features (PCA features) and FoBa was used to fit a model. Maximum Relevancy Minimum Redundancy (mRMR) [39] is a feature selection method which attempts to find features that are maximally relevant to the phenotype and simultaneously the selected features are non-redundant amongst each other. After features are selected, the rrBLUP method was used for GS on the selected features. mRMR can be viewed as a somewhat simplistic univariate filter-based variable selection that ranks each features based on mutual information criterion (note, however, that this univariate ranking criterion also takes into account feature’s redundancy with respect to other features).

Each GS method was evaluated on all data using ten-fold cross validation, i.e. splitting the data into ten evenly sized groups, training on 90%, predicting the remaining 10% data, and computing the correlation of the predictions to the given phenotype values. The square of Person’s correlation of the predicted versus the given phenotypes is called the *coefficient of determination* and denoted *r* ^2^ below. Additionally we performed a round of training and testing on all available data. In the following we present an analysis of the results and discuss possible reasons for the performance of the GS methods with the hope to guide future researchers and perhaps breeders.

## Materials and Methods

### Data Sets

Three empirical data sets and one simulated data set from recent publications were identified as benchmark datasets for this problem.

#### Rice

The asian rice—*Oryza sativa* (O. sativa)—dataset was taken from the supplementary material in [25]. This dataset contains 44,100 SNP variants from 413 accessions of O. sativa, taken from 82 countries and containing 34 phenotypes. The 31,663 tagging SNPs derived from the *Oryza*SNP project as described in the Method section of [25] were used for the genomic selection study here. Among the 34 phenotypes evaluated, only two phenotypes—*pericarp color* and *protein content*—were chosen for testing. The protein content phenotype was selected because the associated GWAS p-value plots (supplementary material [25]) indicated a large number of influential SNPS. In contrast, the pericarp color phenotype was selected as its GWAS p-value plots indicated only a few influential SNPs. Moreover, each trait seemed to have separate genetic mechanisms as there was a correlation of only -0.11 between the two phenotypes. Additionally, monomorphic markers and markers with call rate < 10% were removed for the genomic selection study here, the phenotypes were corrected for populations structure by regressing on a PCA model of the population structure. GS methods in the study were performed on the original phenotypes as well as those corrected for population structure. Missing genotype data was imputed using fastPHASE [40].

#### Pig

The Pig dataset—taken from [26]—is a collection data on male and female pigs born since 2000 consists of 3,534 animals from a single PIC nucleus pig line yielding 52,842 SNPs with five measured traits (phenotypes). Only traits 2 and 4 were randomly selected for study here, each a representative of the two types of traits available. In [26], the genotypes were sequenced from the Illumina PorcineSNP60 chip and full pedigree information was available, which we did not use in this study. In the original study, trait 2 was rescaled by a weighted mean of corrected progeny phenotypes, whereas trait 4 was corrected for environmental factors such as year of birth and location. Additionally, genotypes were filtered for minor allele frequency less than 0.001 and with missing genotypes less than 10%. The original study used AlphaImpute to impute any missing data [41]. For our study we used the data as described above which was given in the supplemental data of the original study [26].

#### QTLMAS

QTLMAS dataset was taken from the QTL-MAS Workshop, which was held on May 17–18, 2010 in Poznań Poland [27]. This dataset consists of 3,226 individuals over five generations (F0–F4) with 20 founders, five male and 15 females. There were two phenotype traits, the first a quantitative trait and the second a binary trait. Note, however, that epistasis was simulated only for Trait 1 but not for Trait 2 in this dataset. Only the first four generations (2,326 individuals) had phenotype records. The genome is approximately 500 million bp with five chromosomes, each 100 million bp. In total, each individual was genotyped for 10,031 biallelic SNPs.

#### Maize

Maize dataset was taken from [28], which consists of two maize diversity panels with 300 *flint* and 300 *dent* lines used for the European CornFed program. This set of lines was aggregated from at least seven sources with the intent of covering “European and American diversity of interest for temperate climate conditions.” After quality control, 261 lines from flint and 261 lines from the dent panel were retained for analysis. Both flint and dent lines were crossed with a tester from an opposite heterotic pool and evaluated for flowering time and biomass production in two adjacent trials in five locations. The three traits recorded were male flowering time (Tass_GDD6), plant dry matter yield (DM_Yield), and dry matter content (DMC). The two panels, flint and dent, were genotyped using a 50k Illumina SNP array, which after removing SNPs with high rate of missing markers and high average heterozygosity, yielded 29,094 and 30,027 SNPs respectively.

### Evaluation of Genomic Selection

Each genomic selection method was evaluated on all four datasets (Rice, Pig, QTLMAS, and Maize) using 10-fold cross-validation and global predictive ability. The *10-fold cross-validation* (10CV) method is the technique of splitting, as close as possible, the samples (individuals) of the dataset into ten evenly sized sets called *folds*. Then, nine of the folds are used to train the genomic selection model and the remaining fold is used to test the predictive ability of the trained model. This procedure is repeated for all ten possible ways of choosing nine training folds and one testing fold. For each of the ten cross validations, the ten predicted phenotype vectors are concatenated and Pearson’s *coefficient of correlation*, denoted by *r*, is evaluated between the vector of all predictions and the vector of the ground truth phenotypes. We then computed the *signed* *r* ^2^, i.e. *r* ^2^ times -1 if the correlation is negative, so that the sign of the correlation is preserved; we will slightly abuse the terminology, and call this score simply *r* ^2^, or the *coefficient of determination*, assuming by default that it is always signed. For each dataset, and each phenotype, the folds were pre-computed and every GS method used the same folds. This ensures that the predictability results are comparable across different GS methods.

Note that we treated the binary trait in QTLMAS similarly to the rest of the traits which were quantitative, i.e. we computed the *r* ^2^ metric directly between the actual binary trait and the real-valued predictions made by our methods. Applying binary thresholding to the latter can be used to obtain the prediction for a binary trait. In general, using binary classifiers instead of thresholded regression techniques for binary trait prediction can sometimes (though not always) yield better classification accuracy; however, the focus of current study was rather on quantitative traits and *r* ^2^ metric.

For *global predictability*, the GS model was trained on all available data and the coefficient of correlation was taken between the predicted phenotype data and the actual ground truth phenotype data, also denoted *r* ^2^.

Some of the GS methods presented in this paper rely on feature selection; that is, these methods utilize a reduced set of markers to build/train the model. For these methods, we compute feature selection stability during 10CV. Feature selection stability is computed as follows. For each of the ten folds, the selected features were recorded. Then, the ratio of the number of features selected during at least *k* = 10, 9, … folds versus the total number of features selected across 10CV was computed. That is, if the ratio was 1.0 for *k* = 10, then all the features selected during the entire 10CV process were selected in each of the 10 training-testing iterations; if the ratio was 0.8 for *k* = 7, then 80% of the features were selected in at least (some) 7 folds out of 10. Note that we count the number of times each feature is selected across the *k* folds separately for each feature, e.g., if one considers two features, each occurring in 8 out of 10 folds, those 8 folds are not necessarily the same (in other words, this *pair* of features does not necessarily occur in 8 out of 10 folds).

Heritability of a trait is interpreted by assuming a natural statistical model of the contribution of the genotype and environment to the trait: Phenotype(Y) = Genotype(G) + Environment(E). The *broad sense heritability* is defined as the ratio of the genetic and phenotype variances, $\sigma_{G}^{2}/\sigma_{P}^{2}$. The genetic variance can further be divided into its additive($\sigma_{a}^{2}$), dominance, and epistatic effects. The *narrow sense heritability* is defined as the ratio of the additive and phenotype variances, $\sigma_{a}^{2}/\sigma_{P}^{2}$. See [42, 43]. In this study, we computed the narrow sense heritability. A GBLUP model and the restricted maximum likelihood method were used to estimate the additive genetic variance $\sigma_{a}^{2}$ and phenotype variance $\sigma_{G}^{2}$.

### Genomic Selection Methods

We give a brief overview of the GS selection methods used in the analysis of the four empirical datasets. We label the fifteen methods BayesA, BayesB, BayesCpi, rrBLUP, Epistasis rrBLUP, Elastic net, Bayesian LASSO, FOBA, ISIS, SVR Linear, SVR Polynomial, SVR Radial, SVR Sigmoid, mRMR, and PCA-FOBA. The order of the methods was chosen to roughly match their similarity to one another if possible.

#### Bayes A, Bayes B, Bayes C*π* (Parametric)

Consider the typical situation for genomic selection, where we have the phenotype $\text{y}\in {\mathbb{R}}^{l}$, and the genotype data $\text{x}\in {\mathbb{R}}^{l\times n}$. The foundational model for Bayes A, Bayes B, and Bayes C*π* can be described by

$$\begin{array}{c} y_{j}=\mu_{j}+\sum_{k=1}^{n}x_{jk}\beta_{k}\delta_{k}+e_{j}, \end{array}$$ (1)

where **x**_*jk*_ is the *k*th marker for individual *j*. Each method differs on the underlying assumptions of *β*, *δ*, and the error **e**. In what follows, let $\sigma_{a_{k}}^{2}$ be the variance of *β*_*k*_, $\sigma_{e}^{2}$ be the variance of the error **e**, and *μ* is the fixed effect. In practice, each method is solved using MCMC, and, more specifically, Gibbs Sampling in case of Bayes A.

**Bayes A** Assumes *δ*_*k*_ = 1 ∀*k*, $\beta_{k}∼N(0,\sigma_{a_{k}}^{2})$ ∀*k*, and ${\text{e}}_{i}∼N(0,\sigma_{e}^{2})$. That is, assume each marker *k* has an effect *β*_*k*_ which has its own variance $\sigma_{a_{k}}^{2}$.

**Bayes B** Assumes *δ*_*k*_ ∼ Bernoulli(*π*) ∀*k*, *π* fixed,

$$\begin{array}{c} \left\{ \begin{array}{cc} \beta_{k}∼N\left(0,\sigma_{a_{k}}^{2}\right) & \text{if}\;\delta_{k}=1\\ \beta_{k}=0 & \text{else} \end{array}\;\forall k,\right. \end{array}$$

and ${\text{e}}_{i}∼N(0,\sigma_{e}^{2})$. That is, assume each marker *k* has a fixed probability *π* of being non-zero. If marker *k* is non-zero then assume it has an effect *β*_*k*_ with variance $\sigma_{a_{k}}^{2}$. Bayes A and B first appeared in [2].

**Bayes C*π*** *δ*_*k*_ ∼ Bernoulli(*π*) ∀*k*, *π* random,

$$\begin{array}{c} \left\{ \begin{array}{cc} \beta_{k}∼N\left(0,\sigma_{a}^{2}\right) & \text{if}\;\delta_{k}=1\\ \beta_{k}=0 & \text{else} \end{array}\;\forall k,\right. \end{array}$$

and ${\text{e}}_{i}∼N(0,\sigma_{e}^{2})$. That is, assume each marker *k* has probability *π* of being non-zero. If marker *k* is non-zero then assume it has an effect *β*_*k*_ with variance $\sigma_{a}^{2}$. Note, there is only a single marker variance, $\sigma_{a}^{2}$, for the entire model. Moreover, the probability *π* of non-zero marker effect is random. Bayes C*π* first appeared in [44].

### rrBLUP (Parametric)

*Ridge regression BLUP* can be described by interpreting Eq (1) as a mixed model equation. Assume *δ*_*k*_ = 1 for all *k*, *μ* is the fixed effect, and *β* and **e** are the random effects. Moreover assume $\beta_{k}∼N(0,\sigma_{a}^{2})$ for all *k* and ${\text{e}}_{i}∼N(0,\sigma_{e}^{2})$. Given data **y** and **x** one can use maximum likelihood or restricted maximum likelihood to estimate $\sigma_{a}^{2}$ and $\sigma_{e}^{2}$ and solve for *μ*, *β*_*k*_, and **e** using Henderson’s mixed model equations. This approach to genomic selection was originally given in [2]. rrBLUP is equivalent to ridge regression (see below), if one takes $\lambda =\sigma_{a}^{2}/\sigma_{e}^{2}$, and was used in genomic selection in [45]. More interestingly, rrBLUP is theoretically equivalent to GBLUP [3–5]. The R package rrBLUP [46] was used to perform rrBLUP analysis labeled ‘rrBLUP’ below.

### Epistasis rrBLUP (Parametric)

Epistasis is the interaction of two or more SNP’s which effect the phenotype of interest. It is the departure of the sum of the marginal effects of each SNP alone. We explored the prediction accuracy of modeling all possible pairwise epistasis using the rrBLUP model. That is, the original set of k SNP features were augmented with an addition *k*(*k* − 1)/2 features where each addition feature was given by the multiplication of all possible pairs of SNP feature. The set of original features and the pairwise epistais features were then modeled exactly as in Eq (1) and rrBLUP method was used to solve. However, the rrBLUP R package was unable to handle such a large input. Therefore, custom software was written to perform rrBLUP, specifically, a specialized parallel linear algebra algorithm to perform the underlying operations of rrBLUP.

### Elastic Net, LASSO, and Ridge Regression (Non-Parametric)

Consider the typical situation for linear regression, where we have the training set $\text{y}\in {\mathbb{R}}^{l}$, $\text{x}\in {\mathbb{R}}^{l\times n}$. In a standard linear regression, we wish to find parameters *β*_0_, ***β*** such that the sum of square residuals, $\sum_{i=1}^{l}{\left(y_{i}-\beta_{0}-x_{i,\cdot }^{⊤}\beta \right)}^{2}$, is minimized.

The *LASSO* approach [47, 48] uses an additional *l*_1_ penalty which aims to achieve a sparse solution. This idea has even been extended to *group LASSO* where variable are included or excluded in groups [49, 50]. Alternatively, *ridge regression* (or *Tikhonov regularization*) [51] uses an *l*_2_ penalty which is ideal for the case when many predictors have non-zero coefficients. *Elastic Net (EN)* uses both an *l*_1_ and *l*_2_ penalty with a trade off parameter between the two [29]. Consequently, LASSO and ridge regression can be seen as special cases of Elastic Net. See [52] and references therein. The Elastic Net problem can be stated as

$$\begin{array}{ccc} & & \underset{\left(\beta_{0},\beta \right)\in {\mathbb{R}}^{n+1}}{min}\;[\frac{1}{2l}\sum_{i=1}^{l}{\left(y_{i}-\beta_{0}-x_{i,\cdot }^{⊤}\beta \right)}^{2}+\lambda P_{\alpha }\left(\beta \right)],\\ & & \;\text{where}\\ & & P_{\alpha }\left(\beta \right)=\left(1-\alpha \right)\frac{1}{2}{∥\beta ∥}_{l_{2}}^{2}+\alpha {∥\beta ∥}_{l_{1}}. \end{array}$$ (2)

Thus, *α* = 1 corresponds to LASSO and *α* = 0 corresponds to ridge. The Elastic Net (with non-zero *α*) can be easily extended for genome-wide association studies by use of the non-zero ***β*** parameters selected when training the data. That is, the *l*_1_ penalty achieves a sparse solution, and in turn signals which variables contribute most when training on the data.

Let us now assume that each column-vector *x*_*i*, ⋅_ is centered to have zero mean (thus, no need for the intercept coefficient *β*_0_) and standardized to have unit variance. Another way to write the Elastic Net problem is to denote *λ*_1_ = 2*lλα* and *λ*_2_ = *lλ*(1 − *α*), then Eq (2) is equivalent to

$$\begin{array}{c} \underset{\left(\beta \right)\in {\mathbb{R}}^{n+1}}{min}\;[\sum_{i=1}^{l}{\left(y_{i}-x_{i,\cdot }^{⊤}\beta \right)}^{2}+\lambda_{1}{∥\beta ∥}_{l_{1}}+\lambda_{2}{|\beta ∥}_{l_{2}}^{2}]. \end{array}$$ (3)

Note that the Elastic Net avoids some drawbacks of LASSO, such as limitations on the number of nonzero coefficients (LASSO cannot select more nonzeros than the number of samples), and a tendency to pick a single representative from a group of highly correlated (and thus jointly relevant or irrelevant) variables—see [29, 53] for more details. Namely, *l*_1_ penalty on the regression coefficients enforces sparsity by “shrinking” some coefficients to zero, while the *l*_2_ penalty removes the limitations on the number of nonzeros and enforces grouping effect, i.e. highly correlated predictors are assigned similar coefficients [29]. This can improve the interpretability of the model, for example, by discovering a group of relevant SNPs instead of just single representative from the group.

For Elastic Net, we use publicly available Matlab code [54] that implements the LARS-EN algorithm of [29]. It takes as an input the *grouping* parameter *λ*_2_ and the *sparsity* parameter that specifies the desired number of selected predictors. Since this number corresponds to a unique value of *λ*_1_, as shown in [53], we will slightly abuse the notation, and denote the sparsity parameter as *λ*_1_, while always interpreting it as the number of selected predictors.

### Bayesian LASSO (Parametric)

Via the Bayesian LASSO [30, 31], the LASSO estimate for linear regression parameters can be interpreted as a Bayesian posterior mode estimate when the regression parameters have independent Laplace (i.e., double-exponential) priors. Gibbs sampling from this posterior is possible using an expanded hierarchy with conjugate normal priors for the regression parameters and independent exponential priors on their variances. A connection with the inverse-Gaussian distribution provides tractable full conditional distributions. Eq (1) can also be used to describe Bayesian LASSO. One assumes *δ*_*k*_ = 1, $\beta_{k}∼N(0,\tau_{k}^{2})$, where $P(\tau_{k}^{2})=\frac{\lambda^{2}}{2}exp(-\lambda^{2}|\tau_{k}^{2}|)$ for all *k* (see Eq 3 in [30]).

### FoBa (Non-Parametric)

Two heuristics that are widely used in practice are forward and backward greedy algorithms. The Forward greedy algorithm (a.k.a. Orthogonal Matching Pursuit (OMP)) consists of greedily picking an additional feature at every step to aggressively reduce the squared error. The backward greedy algorithm trains a full model with all the features, and greedily remove one feature (with the smallest increase of squared error) at a time. Features are removed greedily if their removal does not substantially increase the cost function (i.e. the squared error). Backward steps aim at correcting for mistakes made in earlier forward steps. The backward algorithm can be computationally costly since it starts with all the features and has no theoretical guarantees of success. The FoBa algorithm is a combination of the two, which is based on the forward greedy algorithm and takes backward steps adaptively whenever beneficial. This algorithm is superior to OMP as it can correct mistakes made in earlier steps. It also enjoys theoretical guarantees of correctness. See [32] for more details.

### Support Vector Regression (Non-Parametric)

Support vector machines (SVMs) are a tool in statistics and machine learning for the task of supervised learning used for either classification or regression [33–37]. Here we are interested in the latter case. Following [55], given a training set (**x**_*i*_, *y*_*i*_), *i* = 1, …*l*, where ${\text{x}}_{i}\in {\mathbb{R}}^{n}$ and $y_{i}\in \mathbb{R}$, the goal of *ε*-SV regression (SVR) is to find a “flat” function *f*(**x**) such that *f*(**x**) is at most *ε* deviation from the targets *y*_*i*_, i.e. ∣*f*(**x**_*i*_) − *y*_*i*_∣ ≤ *ε* ∀*i*. In SVR one assumes *f*(**x**) is a *hyperplane* (a higher dimensional extension of a plane), that is, *f*(**x**) = ⟨**w**,**x**⟩ + **b** where ⟨⋅⟩ denotes the dot-product and “flatness” means one seeks a small ‖**w**‖. This goal can be visualized by imagining trying to find a hyperplane in ${\mathbb{R}}^{n}$ with *ε* thickness such that all the data **x**_*i*_ lie in the *ε*-thickened hyperplane. In most cases this is too strict, so each individual *i* is allowed to violate the *ε*-thickened hyperplane by *ξ*_*i*_, albeit with a penalty. Lastly, a *kernel trick* is typically performed where the original data is mapped into another space, often to assist with non-linear data. This entire task is formulated into an optimization framework and training an SVR requires solving

$$\begin{array}{ccc} & & \underset{w,b,\xi }{min}\;\frac{1}{2}w^{⊤}w+C\sum_{i=1}^{l}\xi_{i}\\ & & \;\text{subject}\;\text{to}\;y_{i}\left(w^{⊤}\phi \left(x_{i}\right)+b\right)\geq 1-\xi_{i}-\epsilon ,\\ & & \;\xi_{i}\geq 0. \end{array}$$ (4)

The data vectors **x**_*i*_ are mapped to another space via the function *ϕ*, and SVM attempts to fit the data in this higher dimensional space. The choice of *ϕ*, or, rather, the associated function *K*(*x*, *x*′) = *ϕ*(*x*)*ϕ*(*x*′) referred to as the *kernel function* and has a large impact on the performance of the regression. Note that for many commonly used types of kernels, the function *ϕ* maps into an infinite-dimensional feature space, and is not specified explicitly; instead, it is implicitly given by the corresponding kernel. The de-facto standard SVM software libsvm [56] provides four kernels:

$$\begin{array}{cc} \text{Linear:} & \;u^{⊤}v,\\ \text{Polynomial:} & \;{\left(\gamma u^{⊤}v+r\right)}^{d},\;\gamma >0,\\ \text{Radial:} & \;exp(-\gamma {∥u-v∥}^{2}),\;\gamma >0,\\ \text{Sigmoid:} & \;tanh(\gamma u^{⊤}v+r). \end{array}$$

## Feature Selection Methods

We discuss now the genomic selection methods which use a preliminary round of feature selection.

### Minimum Redundancy Maximum Relevance (Non-Parametric)

A popular criterion for feature selection is *Max-Relevance and Min-Redundancy* (mRMR) [39]. In fact, several of the authors have extended mRMR to work in a transductive manner and showed it can be very accurate at GS [57]. Max-Relevance approach is to search features satisfying the Eq (5), which measures the mean value of all mutual information values between individual feature *x*_*i*_ and class variable *c*.

$$\begin{array}{c} max\;D\left(S,c\right),D=\frac{1}{\left|S\right|}\sum_{x_{i}\in S}I\left(x_{i};c\right) \end{array}$$ (5)

where *S* is the selected feature set, *I*(*x*_*i*_; *c*) is the mutual information between *x*_*i*_ and *c*.

However, feature selection just based on max-relevance tends to select features that have high redundancy, namely the correlation of the selected features tend to be big. If we remove some of the features that are highly correlated with other features, the respective class-discriminative power would not change much. Therefore, Min-Redundancy is proposed to select mutually exclusive features:

$$\begin{array}{c} min\;R\left(S\right),R=\frac{1}{{\left|S\right|}^{2}}\sum_{x_{i},x_{j}\in S}I\left(x_{i},x_{j}\right) \end{array}$$ (6)

An operator Φ(*D*, *R*) is defined to combine *D* and *R* from the above two equations where *D* and *R* are optimized at the same time:

$$\begin{array}{c} max\;\Phi (D,R),\Phi =D-R \end{array}$$ (7)

An incremental search algorithm is proposed to effectively find the near-optimal features defined by Φ(.). The incremental algorithm works as the following: Assuming feature set *S*_*m*−1_ is already generated, which contains *m* − 1 features. The *m*-th feature needs to be selected from the set *X* − *S*_*m*−1_, which maximizes the following objective function:

$$\begin{array}{c} max_{x_{j}\in X-S_{m-1}}[I\left(x_{j};c\right)-\frac{1}{m-1}\sum_{x_{i}\in S_{m-1}}I\left(x_{j};x_{i}\right)] \end{array}$$ (8)

The computational complexity of every single step in this algorithm is *O*(∣*S*∣ × *M*) where ∣*S*∣ is the size of the current target feature set, *M* is the total number of features. Assuming the target feature set is eventually of size *N*, the complexity of this algorithm is $O(\sum_{i=1}^{N}i\times M)$ = $O(\frac{N^{2}}{2}\times M)$.

For genomic selection we combined mRMR with rrBLUP. That is, we first perform a around of feature selection using mRMR. The target size of the selected feature set is determined using cross-validation. That is, we further divided the training set into 10 folds and conducted an internal 10CV to determine the target size. We vary the target size and compute the prediction accuracy for each target size and select the one with the best prediction accuracy. Note, we conduct mRMR only on the training folds in order to select a subset of features and rrBLUP is trained on these selected features. After the top features are selected we then build a GS model using rrBLUP only on the top features. For the prediction of new phenotypes we only use the top features identified in the training round and the previously trained rrBLUP model. Throughout the remainder of the article we will refer to the GS method where we combine mRMR and rrBLUP simply as mRMR.

### Mutual Information Estimation

For two given vectors *X*, *Y*, their mutual information is computed as follows:

$$\begin{array}{c} I\left(X,Y\right)=\sum_{y\in Y}\sum_{x\in X}p\left(x,y\right)log(\frac{p(x,y)}{p(x)p(y)}), \end{array}$$ (9)

where *p*(*x*) is the marginal probability *p*(*X* = *x*) and *p*(*x*, *y*) is the joint probability *p*(*X* = *x*, *Y* = *y*). For vectors with discreet values, we can easily compute *p*(*x*), *p*(*y*), *p*(*x*, *y*) by considering the frequency of the corresponding values. For continuous values, the summation in the above formula should be replaced with integral, as follows:

$$\begin{array}{c} I\left(X,Y\right)=\int_{Y}\int_{X}p\left(x,y\right)log(\frac{p(x,y)}{p(x)p(y)})dxdy. \end{array}$$ (10)

One advantage of the mutual information based method is that in our problem setting, the genotypes are integers with possible value from the set {0, 1, 2}. Therefore, we can use Eq (9) to compute the redundancies among them. We do not need any discretization and thus the estimation is accurate. The phenotypes, or genetic traits, have continuous values. When we compute the relevance between phenotype and genotype, we can approximate Eq (10) with Eq (9) by rounding the continuous phenotype values into integers. However, when the phenotype has values very close to each other for different samples, rounding the values may introduce large error. Instead, we perform discretization of the phenotype value. We first compute the z-score of phenotype value for each sample as $\frac{x-\mu }{\delta }$, where *μ* is the mean and *σ* is the standard deviation. Then, we assign discretized values to samples according to their z-score using the following formula:

$$\begin{array}{ccc} discretized\;value & = & \left\{ \begin{array}{cc} -1 & \;\text{if}\;\text{z-score}\;<\;\text{-1}\\ 1 & \;\text{if}\;\text{z-score}\;>\;\text{1}\\ 0 & \;\text{otherwise} \end{array}\right. \end{array}$$ (11)

### PCA FOBA (Non-Parametric)

PCA (Principle Component Analysis) FOBA is a method where PCA is applied first in a transductive manner then FOBA is applied to accomplish the regression task. As PCA is only dependent on the features, all the training data and unlabeled test data are included. As the number of features is much more than the number of samples, the transductive PCA takes as many features as samples. FOBA is then conducted on the reduced feature set.

### ISIS (Non-Parametric)

ISIS is the iterative form of the Sure Independence Screening (SIS) method developed by [38]. The basic SIS procedure selects a subset of predictors/SNPs according to their correlation with the response. Specifically, given the scaled predictor/SNP matrix *X*, the vector *ω* = *X*′*y* is computed. The top *T* predictors/SNPs with largest component-wise magnitude ∣*ω*_*i*_∣ are selected. For prediction, ridge regression is subsequently applied, using only the subset of predictors/SNPs selected by SIS. For the tests performed in this analysis, we choose *T* = 1000.

ISIS extend SIS as follows. Given a first round of SIS followed with an estimation procedure such as ridge regression, SIS is applied again treating the residuals of the ridge regression as if they were the response. Then ridge regression is reapplied using the union of predictors/SNPs selected in the previous iterations. The procedure is then repeated.

As reflected in its name, SIS enjoys the theoretical guarantee of sure screening, namely with asymptotic probability one, SIS will not discard any relevant predictors.

## Results

**Fig 1:**
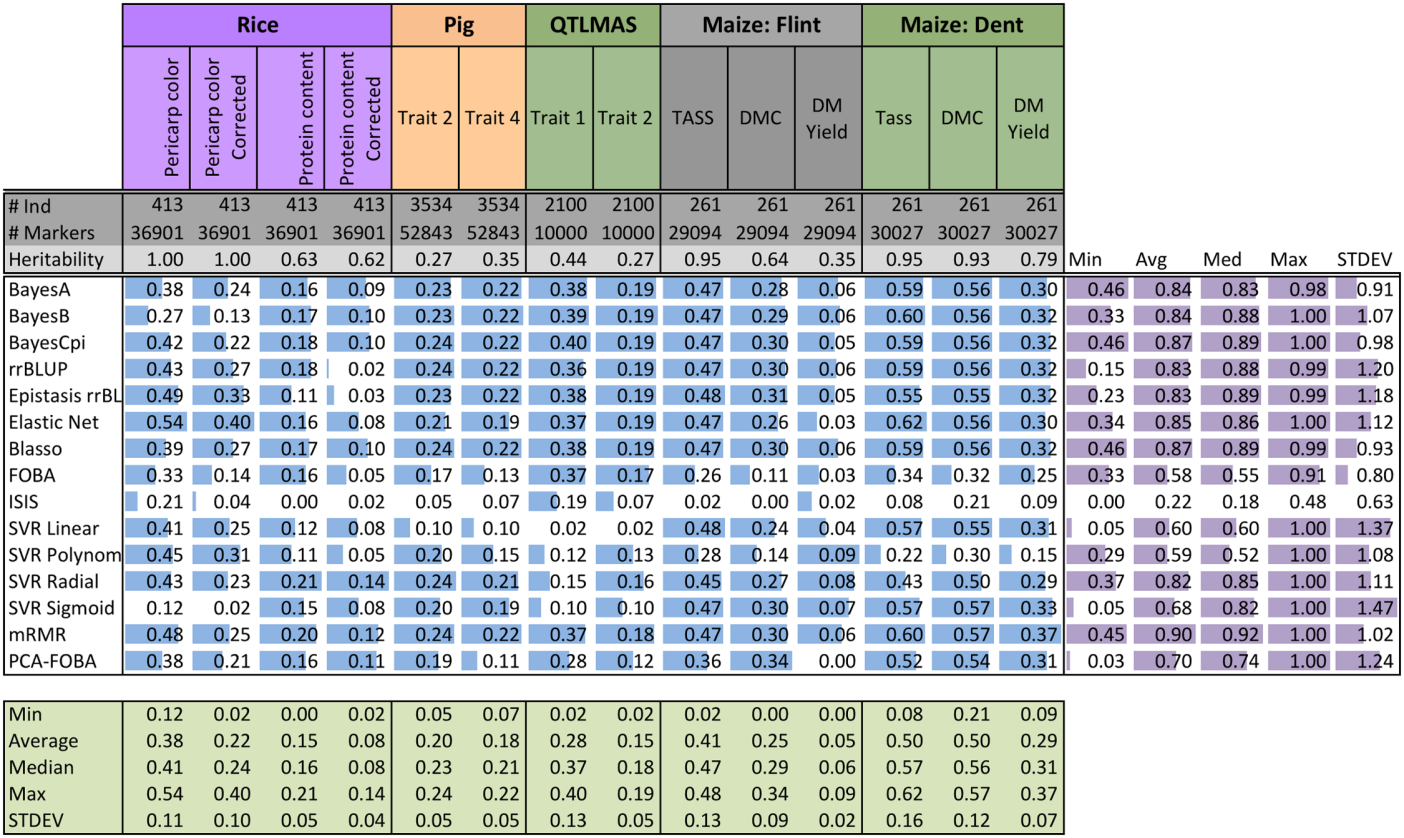
Coefficient of determination (*r* ^2^) of fifteen GS methods on Rice, Pig, QTLMAS, and Maize data under 10-fold cross-validation (10CV). The same folds were used across each data set. Each cell contains the numeric *r* ^2^ score. Additionally, for each dataset (vertical column) bar plots are shown. Bar plots are *normalized* by the minimum and maximum for each data set. Thus, the best (max) *r* ^2^ for a data set will have a full bar while the worst (min) *r* ^2^ will have an empty bar. Summarized to the right are the minimum, average, median, maximum, and the standard deviation of the *normalized* *r* ^2^ scores. Summarized below are the minimum, average, median, maximum, and the standard deviation of the *r* ^2^ scores for each data set. The number of individuals (#Ind), number of markers (#Markers), and the heritability are provided for each data set.

**Fig 2:**
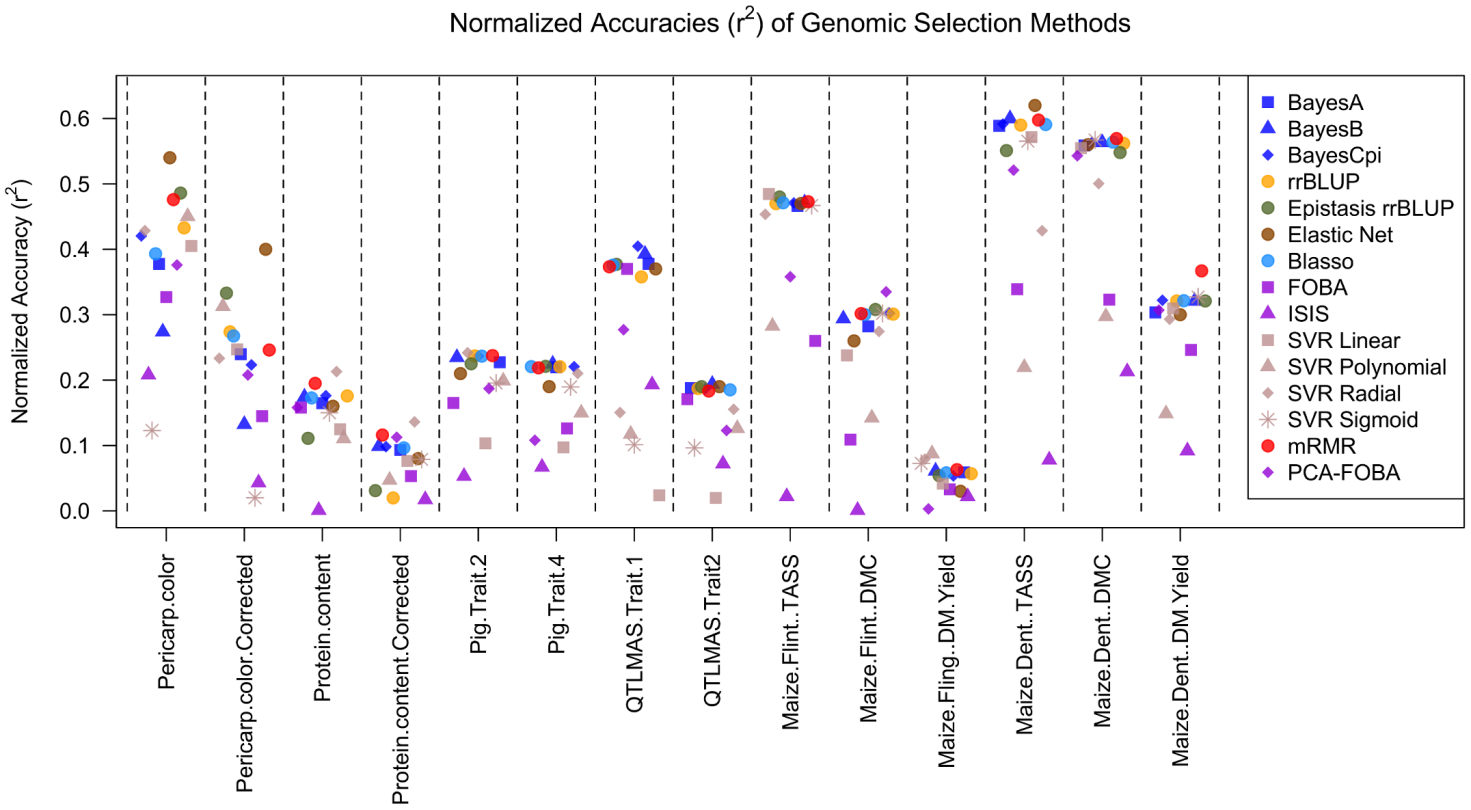
Scatter plot of *normalized* (See Caption in Fig 1) coefficient of determination (*r* ^2^) of fifteen GS methods on Rice, Pig, QTLMAS, and Maize data under 10-fold cross-validation (10CV). The same folds were used across each data set.

**Fig 3:**
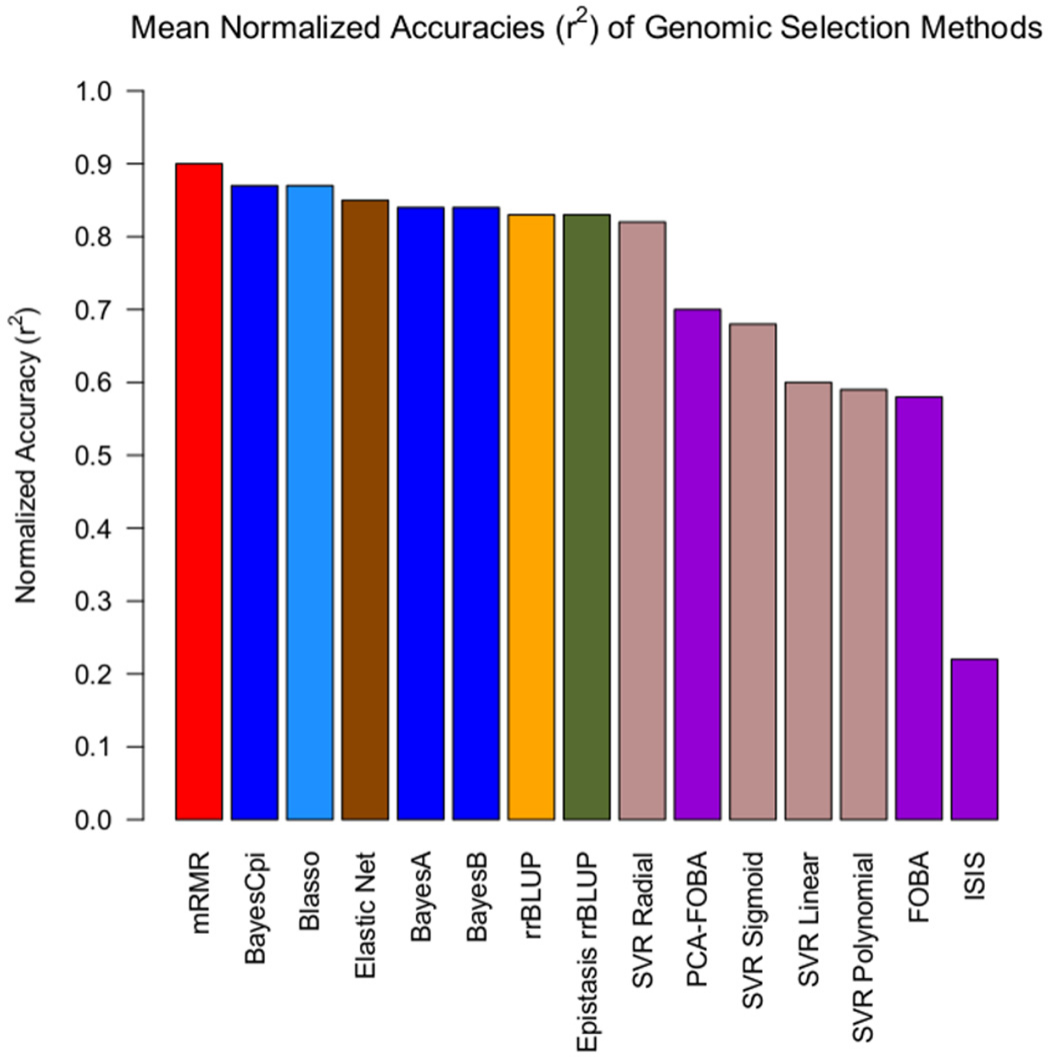
Barplot of mean *normalized* (See Caption in Fig 1) coefficient of determination (*r* ^2^) of fifteen GS methods where the mean was taken over the Rice, Pig, QTLMAS, and Maize data under 10-fold cross-validation (10CV). The same folds were used across each data set.

Fig 1 shows the coefficient of determination *r* ^2^ for the fourteen individual datasets and fifteen genomic selection methods under 10-fold cross-validation. Additionally, Fig 2 visualizes the results from Fig 1 in a form of a scatter plot. Finally, Fig 3 shows a bar plot of the average normalized *r* ^2^, for each of the methods—namely, the results from column *Avg* in Fig 1.

When comparing the overall quality of different methods across all datasets, measured by their normalized average *r* ^2^, as shown in Fig 3, we see that *mRMR is a clear winner among all the methods we tried*, with the highest normalized average *r* ^2^ of 0.90. Moreover, as we can see in Fig 1, mRMR also has the highest median (0.92), and even its worst performance (minimum of 0.45) is among the best worst-case performances across all methods. When looking at the individual datasets, corresponding to columns in Figs 1 and 2, mRMR is the top performer, or among several top-performers, on 8 out of 14 datasets (see the columns 3,4,5,6,8,9, 13,14), and appears well above average, and quite close to the top, on almost all the remaining datasets. These results suggest that, contrary to popular belief, *univariate variable selection* methods such as mRMR *can be well-suited for genomic selection problems*. We also note that the Elastic Net, which is a form of *embedded variable selection*, also performs quite well, even on complex traits, as we show below. Moreover, on the Rice Pericarp color (normal and corrected), the Elastic Net outperforms all other methods. This can be possibly explained by the fact that a relatively few input variables are believed to be responsible for this trait, and thus sparse regression models are well-suited for modeling such data (for more detail, see the Discussion section below).

Next, as it can be clearly seen in Fig 3, as well as in the *Avg* column in Fig 1, there is a large cluster of “second-best” methods, listed here in the decreasing order based on their average performance (normalized *r* ^2^): BayesCpi, Bayesian LASSO (Blasso), Elastic Net, BayesA, BayesB, rrBLUP, Epistasis rrBLUP, and SVR Radial. Note that the average performance of those methods ranges from 0.82 for SVR Radial to 0.87 for Blasso and BayesCpi, as compared to the superior 0.90 average performance of mRMR. On the other hand, the performance within this cluster of methods is considerably better than the best performance of 0.70 for the next best method, PCA-FOBA, as it is evident from Fig 3. Similarly, their median performance ranges from 0.83 for BayesA to 0.89 for Epistasis rrBLUP, as compared to 0.92 median for mRMR.

**Fig 4:**
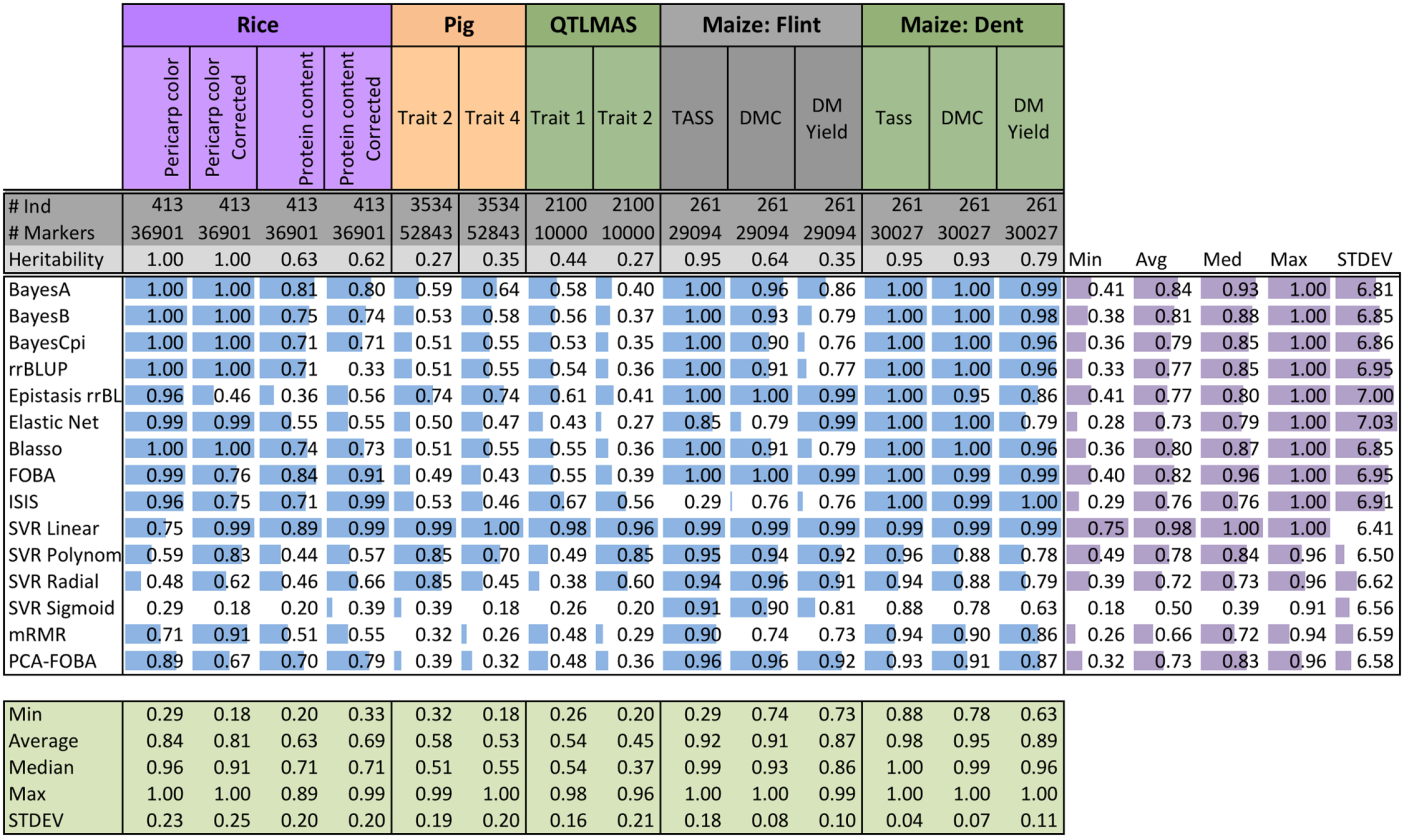
Coefficient of determination (*r* ^2^) of fifteen GS methods on Rice, Pig, QTLMAS, and Maize data where each method was trained on the entire dataset (global). Each cell contains the numeric *r* ^2^ score. Additionally, for each dataset (vertical column) bar plots are shown. Bar plots are *normalized* by the minimum and maximum for each data set. Thus, the best (max) *r* ^2^ for a data set will have a full bar while the worst (min) *r* ^2^ will have an empty bar. Summarized to the right are the minimum, average, median, maximum, and the standard deviation of the *normalized* *r* ^2^ scores. Summarized below are the minimum, average, median, maximum, and the standard deviation of the *r* ^2^ scores for each data set. The number of individuals (#Ind), number of markers (#Markers), and the heritability are provided for each data set.

**Fig 5:**
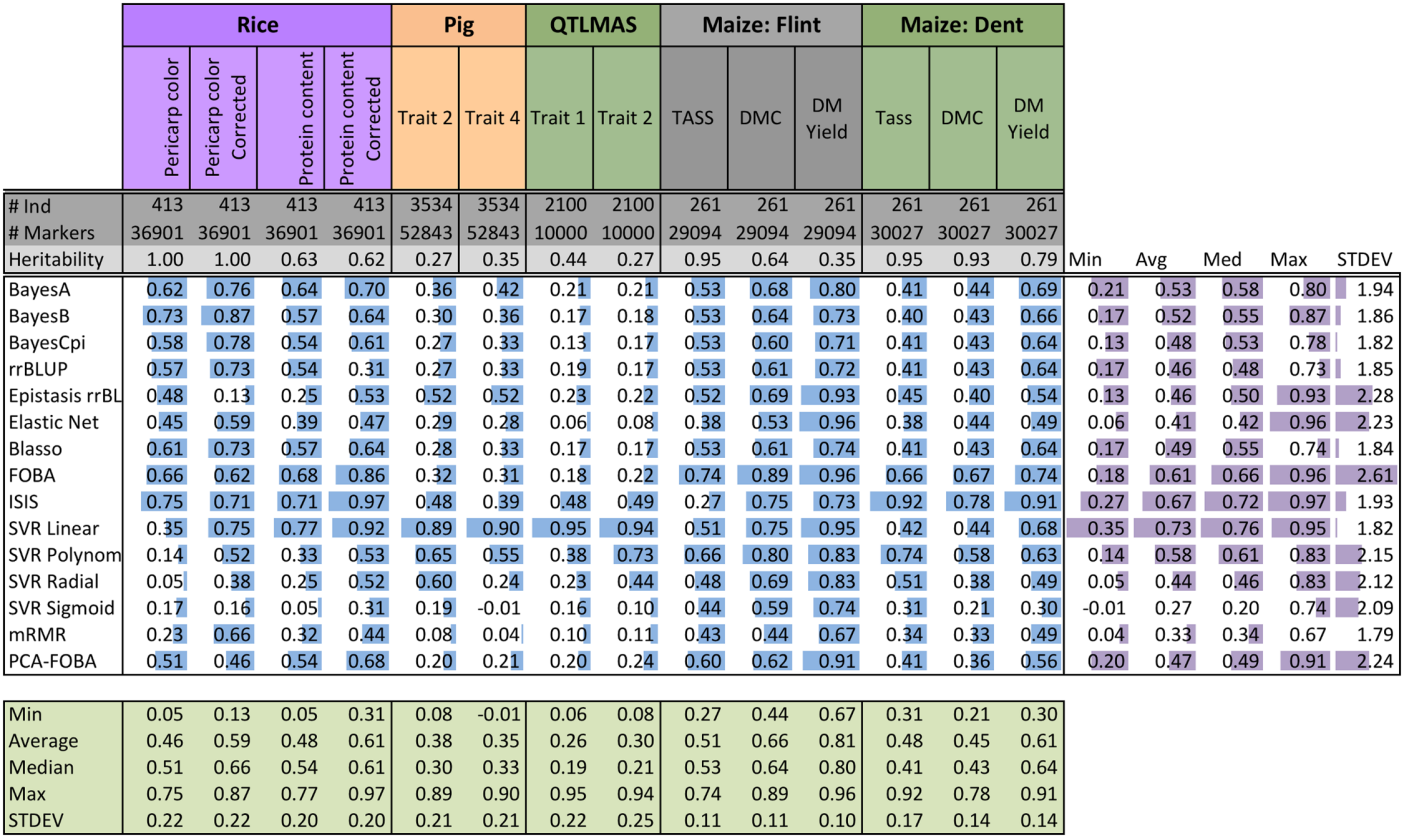
Difference of coefficients of determination (*r* ^2^) for global (each method trained on the entire dataset) and 10CV of fifteen GS methods on Rice, Pig, QTLMAS, and Maize data. Each cell contains numeric difference of 10CV *r* ^2^ and global *r* ^2^. Additionally for each dataset (vertical column) bar plots are shown. Bar plots are *normalized* by the minimum and maximum for each data set. Summarized to the right are the minimum, average, median, maximum, and the standard deviation of the *normalized* difference scores. Summarized below are the minimum, average, median, maximum, and the standard deviation of the difference scores for each data set. The number of individuals (#Ind), number of markers (#Markers), and the heritability are provided for each data set.

Unlike the “second-best” cluster of methods, quite close to the winning performance of mRMR, the “third-best” cluster that includes PCA-FOBA, SVR Sigmoid, SVR Linear, SVR Polynomial and FOBA algorithms, performs considerably worse than the previous group, as we can see from Fig 3. The average performance in this cluster ranges from 0.58 for FOBA to 0.70 for PCA-FOBA, and the median performance ranges from 0.52 for SVR Polynomial to 0.74 for PCA-FOBA, as compared to the lowest median of 0.83 in the second-best cluster. One likely explanation is these models show evidence of overfitting, looking at the difference of coefficients of determination computed using 10CV versus training on the entire data set (global, as shown in Fig 4): see Fig 5 for details.

It is interesting to note that not only SVR Linear, but also SVR Polynomial, and to a somewhat smaller extent SVR Sigmoid, fail to capture predictive structure in the data, while a highly nonlinear SVR Radial performs much better. While we may not be able to fully explain this phenomenon, we speculate that the discrete nature of the input variables may result into such nonlinear structure and/or the Gaussian structure of the Radial kernel more closely matches the data being modeled.

We note that ISIS was completely dominated by the other techniques—its average performance of 0.22 is significantly lower than even the worst performance of the third-best cluster (see Fig 3). Moreover, it was always the least accurate method, for all data sets: as its normalized *r* ^2^ (blue bar in Fig 1) is the lowest among all methods, for all columns in Fig 1. ISIS is somewhat similar to mRMR, except that it does not minimize redundancy within iterations. However, in contrast to mRMR, ISIS is fully supervised. Though ISIS is meant to alleviate the failure of “plain vanilla” SIS with respect to correlated features, ISIS still requires very strong assumptions on the structure of the predictors. ISIS can be viewed as a form of matching pursuit or a greedy algorithm for variable selection [58], and requires stringent conditions on the predictors, such as the so-called restricted isometry property, which are actually stricter than those for LASSO. Also, if *T* is set to 1, then ISIS is similar to a matching pursuit method; namely, it becomes closer to FOBA, though worse, in fact, since there are no backward steps. As we have already seen above, FOBA’s performance is already somewhat poor, which partially explains the failure of ISIS, which is close to a suboptimal variant of FOBA (without the backward step).

Some of the genomic methods used in this analysis were previously compared on maize and barley data in the paper by [14]. For example, [14] used rrBLUP, Bayes C*π*, a variant of Bayes B (wBSR), Elastic Net, and SVR. We note that we observed several trends that are similar to the above work, such as, for example, rrBLUP, Elastic net, Bayes C*π*, and Bayes B performing somewhat similar to each other. Moreover, our analysis of SVR linear did not perform as well in the analysis of [14], most likely due to overfitting as the difference between global and 10CV correlation was large. Also, as discussed above, SVR Linear does not seem to be a natural modeling space for the data, as compared to SVR Radial or SVR Sigmoid.

We note that Epistasis rrBLUP performance varies greatly. For example on QTLMAS Trait 2, it performs very well relative to the other methods; however, on Rice Protein content corrected it performs poorly. Both are polygenic traits, i.e. many influential markers. Thus, Epistasis rrBLUP may be overly sensitive to population structure. Additionally, given the addition of nearly a square factor of the number of parameters with respect to the number of SNPs, one would suspect Epistasis rrBLUP to overfit. Surprisingly, this seems to not be the case considering Figs 4 and 5 showing the coefficient of determination (*r* ^2^) of methods trained on all data (global) and the difference between 10CV and global *r* ^2^. More than likely, the regression parameters used were insufficient to properly model the data; either they were too restrictive or not restrictive enough. Further study should be conducted to explore the usefulness of the new epistasis parameters and how to best choose the regression method and parameters. It is also interesting to note that, contrary to what it is supposed to be modeling, Epistasis rrBLUP performed well on more oligogenic traits, such as the Rice Pericarp color. In this case, it may be that all the pair-wise parameters involving the causal markers have high estimated values. That is, if a marker *X* has high correlation with the trait, then all parameters for pairs *X* × *Y* for all *Y* may have high estimated marker effects. This seems plausible as in the non-epistasis case, rrBLUP searches for solutions where all markers have equal variance and are all small. Thus, if there are relatively few causal markers as we speculate, traditional rrBLUP would want to “push down” the causal marker effects.

We now focus on the comparative analysis of different datasets rather than different methods, i.e. on the column-wise analysis, using the row *Max* in Fig 1 to compare best performances achievable on each of the data sets. We immediately notice that the highest accuracies of *r* ^2^ = 0.57-0.62 (mean *r* ^2^ of 0.50, and median *r* ^2^ = 0.56-0.57) are achieved on the Maize *dent* data, for the two out of three traits: *dent* male flowering time (Tass_GDD6) and dry matter content (DMC), i.e. for those two datasets, genotypic information appears to be the most informative about the trait, i.e. these traits have high heritability. The second-best group of data sets, with respect to the best achievable accuracy, included: the Rice dataset for ‘pericarp color’ trait, where the best accuracy was *r* ^2^ = 0.54 achieved by the Elastic Net, and the mean and median *r* ^2^ across all methods were 0.38 and 0.41, respectively, and the Maize dataset, for both *flint* male flowering time (Tass_GDD6) and *dent* plant dry matter yield (DM_Yield), where the best result of *r* ^2^ = 0.48 was achieved by SVR Linear and by Epistasis rrBLUP, though the mean 0.41 and median 0.47 for the former trait are considerably better than the mean 0.29 and median 0.31 for the latter trait.

**Fig 6:**
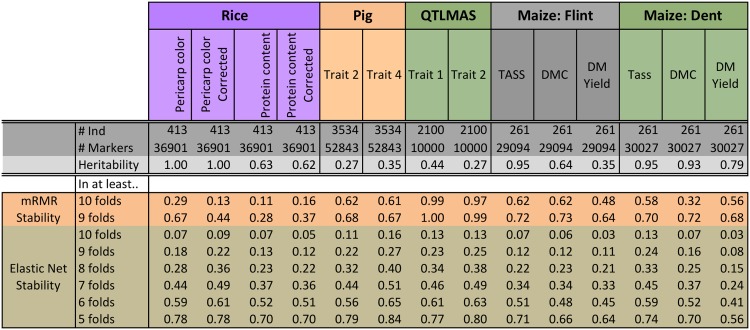
Stability analysis of fifteen GS methods on Rice, Pig, QTLMAS, and Maize data under 10-fold cross-validation (10CV). Reported is the ratio of the number of features selected during at least *k* = 10, 9, … folds versus the total number of features selected across 10CV (note that, for each feature, we count separately the number of folds when the feature is selected). The number of individuals (#Ind), number of markers (#Markers), and the heritability are provided for each data set.

Finally, Fig 6 summarizes feature-selection stability of the two best performing approaches we used, mRMR and Elastic Net. As described earlier, each of the 10CV folds produces a different subset of selected features. For each feature, we compute the number of folds when this feature was selected. Then, for a given number of folds *k*, we computed the number of features selected in at least *k* folds, and divided it by the total number of features selected across 10CV. These ratios are presented in Fig 6, with *k* = 9 and 10 for mRMR, and *k* = 5,6,7,8,9,10 for the Elastic Net. Note that mRMR solutions appear to be considerably more stable then those of the Elastic Net. This is an interesting phenomenon that stems from the nature of each variable-selection method. Recall that the predictor variables, corresponding to SNPs, tend to be correlated with each other, i.e. may form multiple clusters of correlated predictors. If the whole cluster of highly correlated variables is also highly relevant to the target variable, i.e. phenotype, then the original LASSO method—which is practically equivalent to the Elastic Net with sufficiently low values of the grouping parameter *λ*_2_—tends to choose an arbitrary predictor out of such cluster, which can lead to highly unstable solutions. In a sense, this instability is a feature of the data, since in presence of highly correlated predictors there are, indeed, multiple predictive sparse solutions. The Elastic Net attempts to alleviate the instability issues of LASSO in such scenarios, by grouping the correlated variables, i.e. including or excluding them as clusters. However, some of the instability inherent to LASSO-like approaches still remains. On the other hand, mRMR uses a different approach to variable-selection; instead of solving a convex optimization problem, it ranks the variables univariately, based on their individual relevance (mutual information) to phenotype, as well as on their lack of redundancy. Such univariate ranking turns out to be much more stable across the cross-validation folds.

## Discussion

Although it is difficult to make global conclusions using only twelve data sets, given the abundance of data available we still are able to draw some meaningful conclusions in order to guide future researchers and breeders. Our main observation is that, somewhat surprisingly, *the univariate feature selection approach, mRMR, comes out as a clear winner*. It outperformed all other methods on average; moreover, it performed surprisingly well even on complex traits where one expects many markers to have an effect. The mRMR method has an added benefit of providing an interpretable model, pointing out the important markers and their relationship to the trait. Secondly, mRMR was remarkably stable in the selection of its features during each of the 10 folds during cross validation. This is likely due to the fact that mRMR takes into account the information gain that a feature provides about the target variable, while being non-redundant with respect to the other features. Moreover, another variable-selection method—Elastic Net, an example of the so-called sparse regression—also performed quite well overall, though it fell into the second-best group of methods, closely following the winner, mRMR.

**Fig 7:**
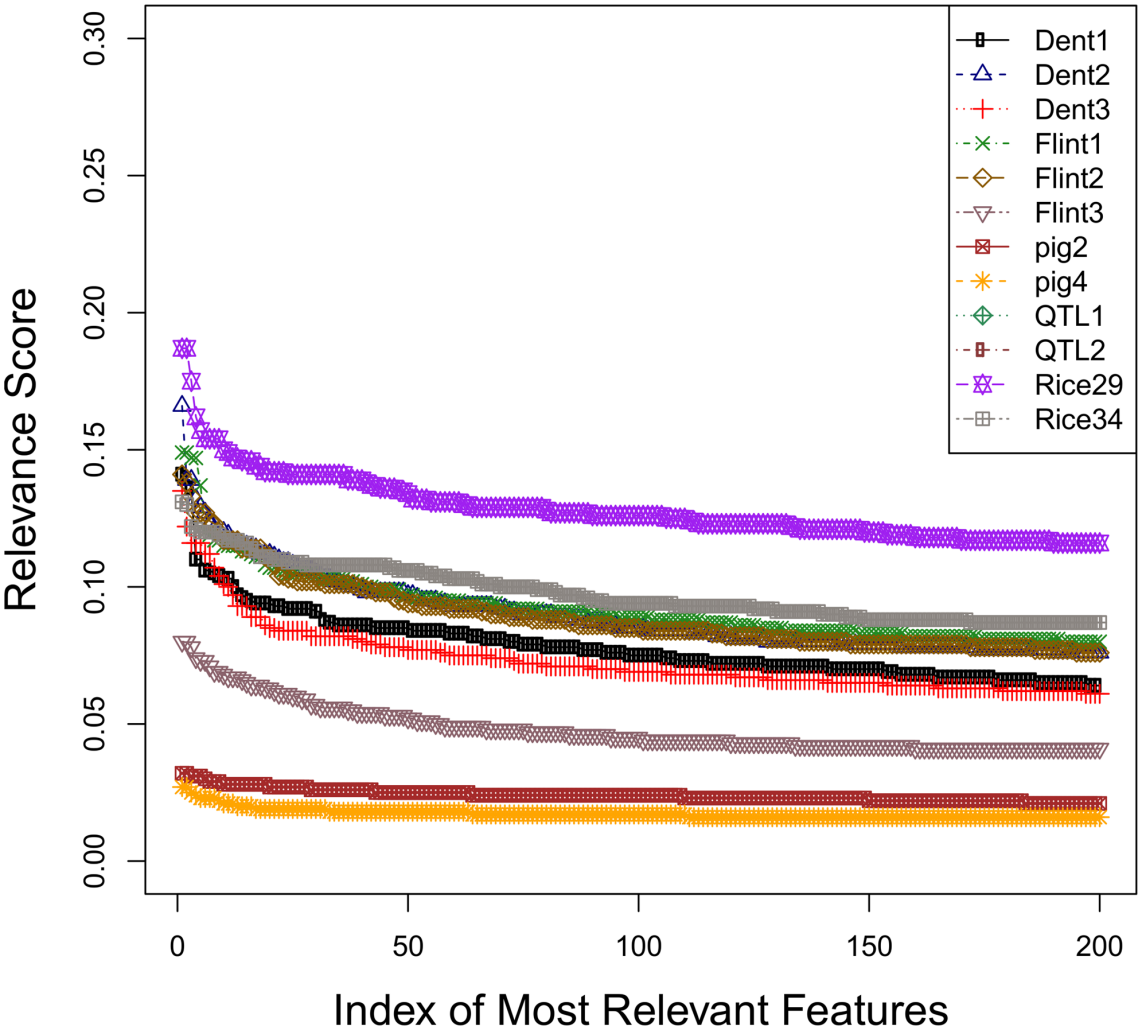
Relevance scores for all features, ranked from the most- to the least-relevant, for each dataset. The correspondence between the legend notation herein and in Fig 2 is as follows: ‘Rice29’ denotes ‘Rice Pericarp color’, ‘Rice34’ denotes ‘Rice Protein content’, ‘QTL1’ and ‘QTL2’ denote QTLMAS traits 1 and 2, respectively, while ‘pig2’ and ‘pig4’ denote the Pig data traits 2 and 4, respectively. Finally, ‘Flint1’, ‘Flint2’ and ‘Flint3’ correspond to the ‘Maize.Flint.TASS’, ‘Maize.Flint.DMC’ and Maize.FlintDM.Yield’, respectively, while ‘Dent1’, ‘Dent2’ and ‘Dent3’ correspond to ‘Maize.Dent.TASS’, ‘Maize.Dent.DMC’, and ‘Maize.Dent.DM.Yield’, respectively.

An intuitive explanation of good performance demonstrated by feature-selection approaches, such as mRMR and the Elastic Net, on specific datasets, can be potentially linked to observations made in the original paper by [47], and reinforced by more recent studies (see, for example, Chapter 3 in [59]). In [47], a simulation study compared univariate feature selection (subset selection regression) versus sparse regression (the LASSO approach) and versus the ridge regression (closely related to rrBLUP). It was observed that the univariate feature selection works best when there is a very small number of large effects, i.e. a small number of predictive variable highly relevant to the target variable. When this number increases to some moderate size, sparse regression performs best. However, neither the subset selection nor the sparse regression appear to work well when there is a large number of small effects, i.e. there is no clear distinction between the relevance of the predictors. In our experiments, mRMR is a more sophisticated version of a univariate feature selection, as it considers both relevance to the target and redundance across the features; the Elastic Net is an augmented version of the original LASSO method, while rrBLUP is closely related to ridge regression. While we do not expect the exact correspondence with the observations made by [47], it is interesting to see somewhat similar behavior. In Fig 7, we plot the relevance scores of different features, ranked from best to worst, for each dataset. Note that for the Rice dataset, Pericarp color trait, there is clearly a small number of highly relevant features, followed by a large number of much less relevant ones, i.e., we have a relatively small number of large effects; note that mRMR and especially the Elastic Net, indeed, works best on this data, outperforming rrBLUP and other competitors, as shown previously in Fig 2. Similar behavior is observed for the Maize.Dent.DM.Yield dataset in Fig 2, shown as Dent 3 plot in Fig 7: a small subset of high-relevance (top-ranked) features, followed by the much lower-relevance majority; mRMR performs best here, clearly outperforming rrBLUP; however, the Elastic Net is also outperformed by mRMR, perhaps due to small (rather than moderate) number of large effects. On the other hand, on the two Pig datasets, practically all features have almost the same relevance scores, i.e. fall into the last category, large number of small effects. Note that on these datasets, mRMR and the Elastic net are, respectively, only as good as, or worse, than rrBLUP.

Overall, that standard rrBLUP approach, while performing fairly well, still fell into the large second-best category of methods, that also included methods such as Blasso, BayesCpi, Elastic Net, BayesA, BayesB, Epistasis rrBLUP, and SVR Radial. The remaining methods, including PCA-FOBA, SVR Sigmoid, SVR Linear, SVR Polynomial and FOBA algorithms, performs considerably worse than the second-best group, and ISIS appears to be particularly inaccurate on the data sets we considered.

Finally, it still remains to be explored what data properties are essential for a specific method to work well (or poorly). One hypotheses we propose is that the discrete, rather than continuous, nature of the genomic data, where the variables are ternary, with a highly skewed distribution towards two out of three values being most frequent, may play an important role, affecting performance of some of the methods.
